# Allelopathic Potential and Active Substances from *Wedelia Chinensis* (Osbeck)

**DOI:** 10.3390/foods9111591

**Published:** 2020-11-02

**Authors:** Kawsar Hossen, Krishna Rany Das, Shun Okada, Arihiro Iwasaki, Kiyotake Suenaga, Hisashi Kato-Noguchi

**Affiliations:** 1 Department of Applied Biological Science, Faculty of Agriculture, Kagawa University, Miki, Kagawa 761-0795, Japan; kwsarbau@gmail.com (K.H.); k_das007@yahoo.com (K.R.D.); oskhaudna.30@gmail.com (S.O.); 2 The United Graduate School of Agricultural Sciences, Ehime University, 3-5-7 Tarumi, Matsuyama 790-8566, Japan; 3 Department of Entomology, Faculty of Agriculture, Bangladesh Agricultural University, Mymensingh 2202, Bangladesh; 4 Department of Chemistry, Faculty of Science and Technology, Keio University, 3-14-1 Hiyoshi, Kohoku, Yokohama 223-8522, Japan; a.iwasaki@chem.keio.ac.jp (A.I.); suenaga@chem.keio.ac.jp (K.S.)

**Keywords:** *Wedelia chinensis*, organic farming, phytotoxic substances, vanillic acid, gallic acid

## Abstract

*Wedelia chinensis* (Asteraceae) is a wetland herb native to India, China, and Japan. It is a valuable medicinal plant recorded to have pharmaceutical properties. However, the phytotoxic potential of *Wedelia chinensis* has not yet been examined. Thus, we carried out this study to establish the allelopathic effects of *Wedelia chinensis* and to identify its phytotoxic substances. Extracts of *Wedelia chinensis* exhibited high inhibitory activity against the root and shoot growth of cress, alfalfa, rapeseed, lettuce, foxtail fescue, Italian ryegrass, timothy, and barnyard grass. The inhibition was varied with species and was dependent on concentrations. The extracts were separated through several purification steps, and the two effective substances were isolated and characterized as vanillic acid and gallic acid using spectral analysis. Vanillic acid and gallic acid significantly arrested the growth of cress and Italian ryegrass seedlings. The concentrations of vanillic acid and gallic acid needed for 50% inhibition (I_50_ values) of the seedling growth of the cress and Italian ryegrass were 0.04–15.4 and 0.45–6.6 mM, respectively. The findings suggest that vanillic acid and gallic acid may be required for the growth inhibitory activities of *Wedelia chinensis*.

## 1. Introduction

Organic farming emerged at the beginning of the 20th century as an alternative agricultural method to demote the hazardous effect of nonnatural herbicides on the environment and people [1,2]. Natural substances are used in organic farming, and at the same time, the use of synthetic substances is banned or severely restricted [3]. Organic farming needs various types of agricultural crop that help to sustain beneficial microorganisms in the soil and to enhance soil conservation to increase farm production. Accordingly, weeds should be controlled without applying chemical herbicides that cause enormous problems [4]. Thus, searching for nature-based product alternatives to synthetic herbicides is now a pressing issue to control weeds, which is a major impediment to crop production [5,6]. In light of these concerns, allelopathy can be explored and used as alternative weed management over synthetic herbicides [7]. Many studies have reported on the use of phytotoxic substances as a nature-friendly approach to weed management [7,8,9].

The perennial herb *Wedelia chinensis* from the Asteraceae family is usually named Wedelia in Chinese, bhringraj in Hindi, and manjal karisalanganni in Tamil [10]. *Wedelia chinensis* is a climbing herb that is introduced in submerged areas in Assam, Uttar, and Andhra Pradesh and in offshore areas of India. It is also grown in the Madras Presidency of India, Japan, and China [11,12]. The herb is a fragile, expanding, and hairy-type plant, with branches usually up to 50 cm long.

The leaf architecture is simple, arranged oppositely with subsessile leaflets and short, white hairs. Its flower is an axillary head and yellow. The fruits are nearly oval with hairs on the surface [13,14,15]. The literature reports that different parts (leaves, stems, and roots) of *Wedelia chinensis* are applied as hepatoprotection and a cholagogue and as folk medicine to treat various diseases such as diarrhea, jaundice, cough, diphtheria, headache, and pertussis; to help relieve mental stress; and to promote sleep [16,17]. Conventionally, the leaves, stems, and fruits are used in childbirth and to treat bites and stings, kidney dysfunction, fever, amenorrhea, infection, and wounds [18]. Leave extracts are a natural way to generally apply anti-inflammatory medicines such as Dolonex (Piroxicam) Brufen, and Voveran [13].

Pharmacological investigations have revealed that *Wedelia chinensis* had effects on diterpenoids, sesquiterpenes, triterpenoids, flavonoids, organic acids, and steroids [19,20] and has antioxidant [21,22], anti-inflammatory and antimicrobial [23], and anticancer effects [24]. Moreover, *Wedelia chinensis* is a common garden herb that tends to form a community, and field investigations have shown that there are few weeds in its community [25]. Although *Wedelia chinensis* is known to contain many pharmaceutical features, the allelopathic effects of this plant have not yet been recorded in the literature. Therefore, the study was conducted to explore the allelopathy and to identify the allelochemicals from *Wedelia chinensis*.

## 2. Materials and Methods

### 2.1. Plant Material Collection

The *Wedelia chinensis* is a perennial climbing-type herb that is grown in the submerged and seashore areas of subtropical regions. The whole plants (except roots) of *Wedelia chinensis* were collected from the Botanical Garden of Bangladesh Agricultural University (BAU), Mymensingh, Bangladesh in August and September 2016. The species was identified by Sarwar Abul Khayer Mohammad Golam (Department of Crop Botany, BAU) at voucher number BGBAU 16MP-0003 deposited in the Medicinal Plant Herbarium, Botanical Garden, BAU. All of the plant parts were cleaned under running water, dried in the shade to prevent scorching from sunshine, and then ground into powder with a grinder. The plant powder was put into a polybag and kept at 2 °C before extraction.

### 2.2. Test Plant Species

Eight test plants (including both crop and weed species) were used in this experiment for biological assay: lettuce (*Lactuca sativa* L.), alfalfa (*Medicago sativa* L.), cress (*Lepidium sativum* L.), rapeseed (*Brassica napus* L.), Italian ryegrass (*Lolium multiflorum* Lam.), barnyard grass (*Echinochloa crus-galli* (L.) P. Beauv.), foxtail fescue (*Vulpia myuros* (L.) C.C. Gmel.), and timothy (*Phleum pratense* L.). These species were selected based on their noted growth pattern (alfalfa, lettuce, cress, and rapeseed), susceptibility to allelopathic substances, and abundance (Italian ryegrass, foxtail fescue, timothy, and barnyard grass) in crop fields as weeds.

### 2.3. Extraction

The *Wedelia chinensis* powder (1.60 kg) was extracted utilizing 8 L of 70% (*v/v*) aqueous methanol. The plant extracts were filtered onto a single layer of filter paper (No. 2; Toyo Roshi Ltd., Tokyo, Japan). Plant residues were extracted again for one day with the equivalent amount of methanol (100%) and filtered. Both filtrates were mixed and evaporated until dry using a rotary evaporator at 40 °C.

### 2.4. Growth Bioassay

The extracts of *Wedelia chinensis* were diluted into 300 mL of methanol to make six test concentrations as 1, 3, 10, 30, 100, and 300 mg DW (dry weight) equivalent extract/mL. Aliquots of the extract were applied to the single sheet of filter paper (No. 02) into Petri dishes (28 mm) at assay concentrations and kept in a draft chamber to desiccate the methanol. The filter paper was then soaked with 0.6 mL (*v/v*) aqueous solution of Tween 20 (polyoxyethylene sorbitan monolaurate; Nacalai, Kyoto, Japan) in each Petri dish to serve as a surfactant. Ten seeds of lettuce, alfalfa, cress, and, rapeseed, and 10 pre-emergence seeds of timothy, Italian ryegrass, barnyard grass, and foxtail fescue (incubated in dark condition for 68 h, 48 h, 46 h, and 72 h, respectively, at the temperature of 25 °C) were placed onto the filter paper in the Petri dishes. The control treatments were prepared for the seeds and pre-emergence seeds with an aqueous mixture of polyoxyethylene sorbitan monolaurate without the extracts. After two days of incubation in dark condition at 25 °C, the growth of the all tested plants were estimated. The seedling percentage length of seedlings was determined based on the length of the control seedlings. The concentration needed for 50% growth inhibition (I_50_ values) was determined for each species using a logistic regression equation of the concentration–response curves. Throughout this experiment, the assay was performed with three replications for each model plant and replicated twice (10 seeds or pre-germinated seeds/replication).

### 2.5. Extract Partitions

The *Wedelia chinensis* extracts were evaporated at 40 °C with a rotary evaporator to make an aqueous residue, and pH of the residues was modified to 7.0 by using 1 Molar (M) phosphate buffer. The plant extracts were then partitioned eight times with an equivalent amount of EtOAc (ethyl acetate) and divided into H_2_O (aqueous) and EtOAc fractions. A bioassay was set with cress seeds to measure the phytotoxic effects of H_2_O and EtOAc fractions.

### 2.6. Isolation and Purification of the Active Substances

**Figure 1:**
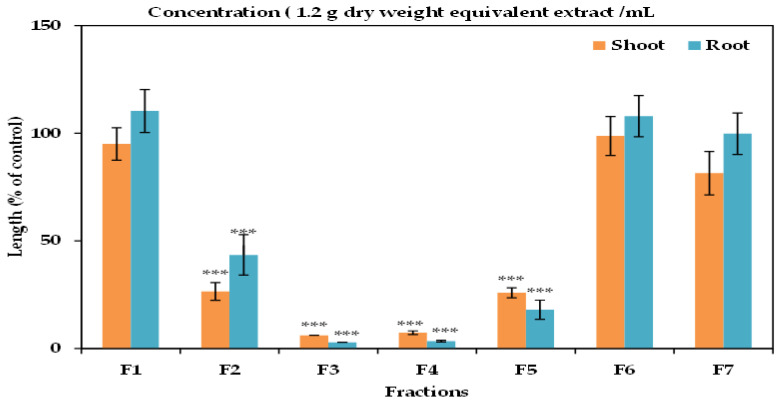
Effect on the seedling growth of cress of all fractions of the extracts of *Wedelia chinensis* that has been obtained from the column of Sephadex LH-20: cress seeds were exposed to the concentration equivalent of the extracts gained from 1.2 g DW (dry weight) of *Wedelia chinensis*/mL for F1 (20% aqueous methanol), F2 (30% aqueous methanol), F3 (40% aqueous methanol), F4 (50% aqueous methanol), F5 (60% aqueous methanol), F6 (80% aqueous methanol), and F7 (methanol). The values are mean ± SE for each treatment from the two independent experiments with 10 seedlings. Asterisks show major variations between treatments and control: *** *p* < 0.001 (ANOVA one way and least significant difference (LSD) test post hoc).

After saturating overnight with sodium sulphate, the EtOAc (ethyl acetate) fraction was evaporated to dryness. The extract residues were loaded into a silica gel column (60 g of silica gel 60, 70–230 mesh; Nacalai Tesque, Kyoto, Japan) and eluted with *n*-hexane with increasing quantities of EtOAc (increased 10%/step (*v/v*), in 150 mL/step) and 300 mL methanol. The repressing effects of all fractions were calculated through the cress assay, as previously stated. In the column of silica gel, the most active fractions were obtained from 60, 70, and 80% EtOAc in *n*-hexane and EtOAc, which were mixed together and then evaporated until complete dryness; purified through a column of Sephadex LH-20 (GE Healthcare Bio-Science AB, SE-75184, Uppsala, Sweden); and rinsed with 20, 30, 40, 50, 60, and 80% (*v/v*) aqueous methanol (150 mL/step) and 300 mL methanol. The highest active fraction was eluted with 40% aqueous methanol (Figure 1), then evaporated until dry, diluted in 20% (*v/v*) aqueous methanol, and burdened into a reverse-phase C_18_ cartridge (YMC Co. Ltd., Kyoto, Japan). The C_18_ cartridge was rinsed with 20, 30, 40, 50, 60, and 80% (*v/v*) aqueous methanol and cold methanol. The highest active fraction was rinsed with 50% aqueous methanol, which was then refined with reverse phase high-performance liquid chromatography (HPLC) (5 µm, 4.6 × 250 mm I.D., Inertsil^®^ ODS-3; GL Science Inc., Tokyo, Japan). The HPLC column was rinsed with 20% (*v/v*) aqueous methanol at a flow rate of 0.8 mL/min. The active substances were determined at the wavelength of 220 nm and at the oven temperature of 40 °C from 62 to 100 min retention time as a colorless substance (substance 1) and from 65 to 70 min retention time as a whitish substance (substance 2). Both substances were characterized using high resolution electrospray ionisation mass spectrometry (HRESIMS), proton nuclear magnetic resonance (^1^H-NMR), and specific rotation.

### 2.7. Bioassay of the Isolated Substances

The extracted compounds were diluted in methanol to make final bioassay concentrations: 0.003, 0.01, 0.03, 0.1, 0.3, and 1 mM of substance 1 and 0.03, 0.1, 0.3, 1, 3, 10, and 30 mM of substance 2 were applied to filter paper (No. 2, 28 mm; Toyo Roshi Ltd., Tokyo, Japan) in Petri dishes (28 mm) and kept in a draft chamber to desiccate the solvent. The growth inhibitory activity of the isolated compounds, as previously described, were calculated using cress and Italian ryegrass. The I_50_ values for these compounds against the tested plant species were determined as mentioned above.

### 2.8. Statistics

Each assay experiment was conducted with three replications and replicated twice in a completely randomized block design. The resulting data were analyzed using SPSS software version 16.0 (SPSS Inc., Chicago, IL, USA). The data that were obtained from each experiment were then subjected to analysis of variance (ANOVA), and the significant differences between the mean of treatments and control were calculated using a post hoc Tukey’s test with least significant difference (LSD) test at 5% level of probability.

## 3. Results

### 3.1. Allelopathic Effects of Wedelia chinensis on the Seedling Growth of the Tested Plant Species

**Figure 2:**
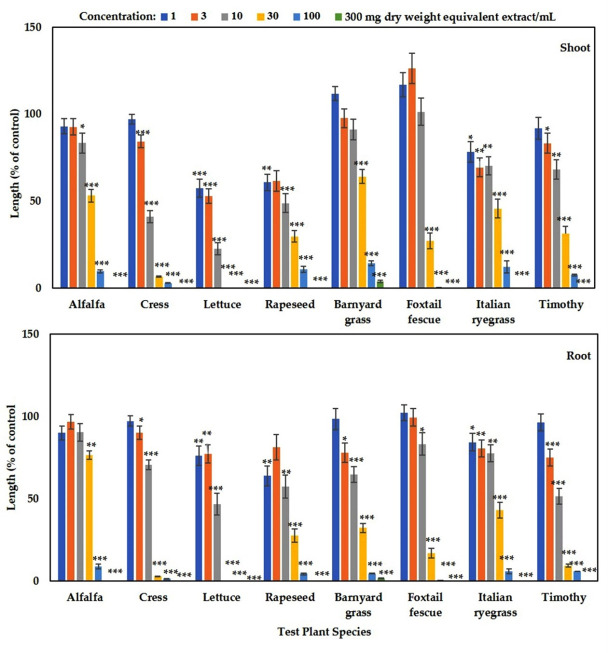
Growth inhibitory effects of *Wedelia chinensis* plant extracts on the shoot and root growth of lettuce, alfalfa, cress, rapeseed, barnyard grass, timothy, Italian ryegrass, and foxtail fescue: the tested species were exposed to the concentrations of 1, 3, 10, 30, 100, and 300 mg DW equivalent extracts of *Wedelia chinensis*/mL. The values are mean ± SE for each treatment from the two independent experiments with 3 replications (10 seedlings for each replication) for every experiment (*n* = 60) that is displayed. Asterisks signify important variations between treatments and control: * *p* < 0.05, ** *p* < 0.01, and *** *p* < 0.001 (ANOVA one-way and LSD test by post hoc).

The aqueous methanol extracts of *Wedelia chinensis* suppressed the seedling growth of the tested plants (lettuce, cress, rapeseed, alfalfa, barnyard grass, Italian ryegrass, timothy, and foxtail fescue) at various concentrations (Figure 2). The growth seedlings of all the tested plants (other than barnyard grass) were completely arrested (100%) in the concentration of 300 mg DW equivalent extract of *Wedelia chinensis*/mL. When the tested plants were exposed to the concentration of 100 mg DW equivalent extract of *W. chinensis*/mL, the lettuce seedlings were completely inhibited and the shoot and root growth of alfalfa, rapeseed, cress, barnyard grass, Italian ryegrass, timothy, and foxtail fescue were restricted to 9, 2.9, 6, 10.6, 12.1, 14.3, 7.4, and 0.2% growth compared with the control shoots and 8.7, 1.3, 4.1, 4.4, 0.1, 5.7, and 5.6% growth compared with the control roots, respectively. The growth inhibition of the tested plants was different at other concentrations (Figure 2). These findings indicated that the *Wedelia chinensis* extracts suppressed the seedling growth of both the dicots and monocots species, and the inhibition of growth increased when the concentration of the extracts increased. The I_5_*_0_* values varied at 3.3–42.2 (for shoots) and 8.7–48 mg (for roots) DW equivalent extract/mL for tested species (Table 1). The lettuce seedling was the most susceptible to the extracts based on I_50_ values, and the least sensitive was the barnyard grass shoots and alfalfa roots. These findings suggest that the growth inhibition by the *Wedelia chinensis* extract varied among the tested plants. The concentration-dependent and species-specific inhibitory effects of *Wedelia chinensis* extracts indicate that this plant might contain an allelopathic potential and might therefore possess allelochemicals.

### 3.2. Identification of the Phytotoxic Substances

**Figure 3:**
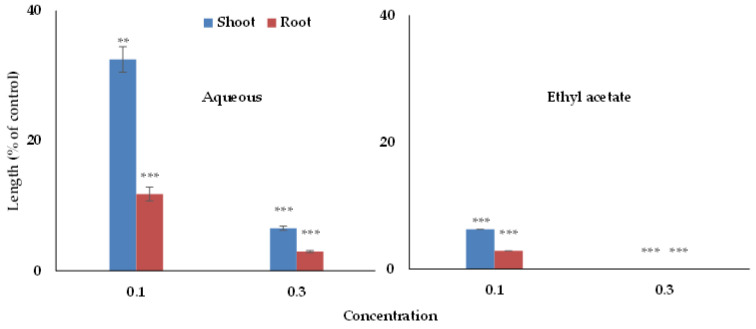
Effect on the seedling growth of cress of the aqueous and ethyl acetate fractions that has been obtained by partitioning of *Wedelia chinensis* extracts: cress was subjected to concentrations equal to 0.1 and 0.3 g DW extracts of *Wedelia chinensis*/mL. The values are mean ± SE for each treatment from the two independent experiments with 10 seedlings. Asterisks show major variations between treatments and control: ** *p* < 0.01 and *** *p* < 0.001 (ANOVA one way and LSD test post hoc).

In the partitioning purification step, the H_2_O and EtOAc fraction of the *Wedelia chinensis* extracts displayed concentration-dependent growth inhibitory effects against the growth of cress seedlings, but a higher inhibitory effect was found in the EtOAc fraction (Figure 3). Thus, the EtOAc fraction was again purified using various chromatographic proceedings (column of silica gel, Sephadex LH-20 column, and reverse-phase C_18_ cartridges), and for each purification proceeding, the inhibitory activity was measured using a cress bioassay. Using the reverse-phase of HPLC in the bottommost purification step, two phytotoxic compounds were isolated. The phytotoxic substances were identified based on NMR and spectral analysis of the data and by comparison with the previously recorded data.

**Figure 4:**
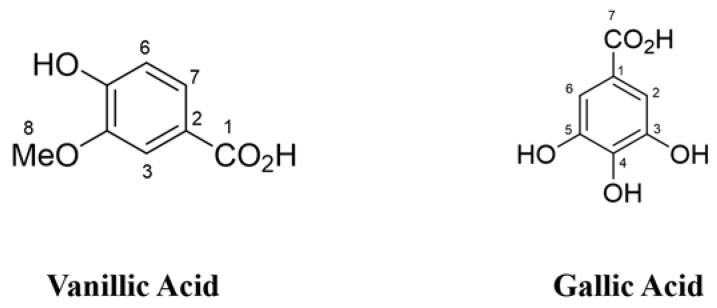
The chemical structures of vanillic acid and gallic acid obtained from the extracts of *Wedelia chinensis*.

The molecular formula of substance 1 was determined as C_8_H_7_O_4_ using HRESIMS *m/z* 167.0348 [M-H]^−^ (calcd for C_8_H_7_O_4_, 167.0348, ∆ = +0.4 mmu); ^1^H NMR (400 MHz, CD_3_OD) δ_H_ 7.56 (d, *J* = 1.6 Hz, 1 H, H3), 7.55 (dd, *J* = 9.4, 1.6 Hz, 1 H, H7), 6.83 (d, *J* = 9.4 Hz, 1 H, H6), 3.89 (s, 3 H, H8). Substance 1 was identified as vanillic acid (Figure 4) by comparing the data with those of the previously reported in the literature [26].

The molecular formula of substance 2 was determined as C_7_H_5_O_5_ using HRESIMS *m/z* 169.0749 [M-H]^−^ (calcd for C_7_H_5_O_5_, 169.0749); ^1^H NMR (400 MHz, CD_3_OD) δ_H_ 7.05 (s, 2 H, H2, 6); ^13^C NMR (100 MHz, CD_3_OD) δ_C_ 170.4 (C–7), 146.4 (C–3, 5), 139.6 (C–4), 122.0 (C–1), 110.3 (C–2, 6). Substance 2 was identified as gallic acid (Figure 4) by comparing the data with those of the previously reported in the literature [27].

### 3.3. Biological Activity of the Isolated Substances

**Figure 5:**
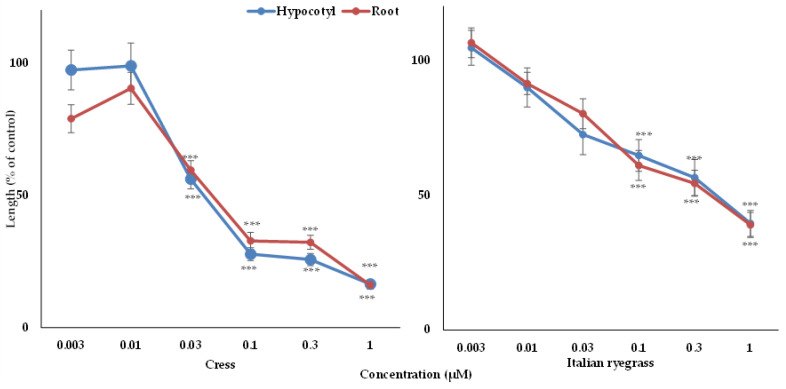
Effect of vanillic acid on the seedling’s growth of cress and Italian ryegrass: mean ± SE from the two separate experiments with three replications (10 seedlings for each replication) for every experiment that is displayed. Asterisks signify important variations between treatments and control: *** *p* < 0.001 (ANOVA one-way and LSD test by post hoc).

**Figure 6:**
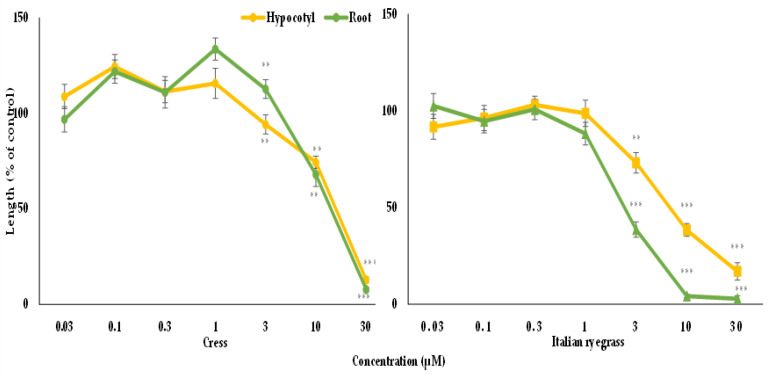
Effect of gallic acid on the seedling’s growth of cress and Italian ryegrass: mean ± SE from the two separate experiments with three replications (10 seedlings for each replication) for every experiment that is displayed. Asterisks signify important variations between treatments and control: ** *p* < 0.01, and *** *p* < 0.001 (ANOVA one-way and LSD test by post hoc).

The biological activity of vanillic acid and gallic acid were tested against cress and Italian ryegrass. The obtained results of the assay exhibited that the growth of the cress and Italian ryegrass seedlings was significantly impaired by both compounds (Figure 5 and Figure 6). The level of inhibition by the isolated compounds increased with increasing concentration, suggesting that the inhibition was dose dependent. Vanillic acid and gallic acid significantly restricted the growth of the cress seedlings at concentrations of 0.03 and 3 mM, respectively (Figure 5 and Figure 6). Vanillic acid had the highest inhibition (12% of control) against the growth of the cress shoots and roots at a concentration of 1 mM, whereas gallic acid exhibited maximum suppression of the growth of the cress shoots and roots at 12.8 and 7.8% of control growth, respectively, at a concentration of 30 mM (Figure 5 and Figure 6). In contrast, vanillic acid and gallic acid significantly arrested the seedling growth of Italian ryegrass at concentrations of 0.1 and 3 mM, respectively (Figure 5 and Figure 6). At 1 mM, vanillic acid showed maximum inhibition against the growth of the Italian ryegrass shoots and roots at 39.4 and 38.9% of control, respectively, while gallic acid displayed maximum suppression against the growth of the shoots and roots at 16.9 and 2.8%, respectively, compared with control growth at a concentration of 30 mM.

The I_50_ values of vanillic acid against cress shoot and root were 0.04 and 0.05 mM, respectively, (Table 2) which are about 12- and 9-times higher, respectively than that for the Italian ryegrass shoots (0.47 mM) and roots (0.45 mM). In contrast, the I_50_ values of gallic acid against Italian ryegrass shoot and root were 6.6 and 2.3 mM, respectively, (Table 2) which are roughly 2.3- and 6-times lower, respectively, than that for the cress shoot (15.4 mM), and root (13.8 mM). The I_50_ values show that vanillic acid had greater growth inhibitory effects against both tested species compared with gallic acid. In addition, the cress shoot and root displayed greater susceptibility to vanillic acid than that of Italian ryegrass, and the Italian ryegrass showed higher sensitivity to gallic acid than cress.

### 3.4. Discussion

The present study showed the phytotoxic activities of aqueous methanol extracts of *Wedelia chinensis*, which significantly restricted the seedlings growth of eight tested species: lettuce, alfalfa, cress, rapeseed, barnyard grass, Italian ryegrass, timothy, and foxtail fescue (Figure 2). Phytotoxic effects increased with increasing extract concentration. Our results corroborate other research studies [28,29,30,31,32,33], which reported the concentration-dependent and species-specific growth inhibitory activities of various plant extracts. These findings suggested that the growth inhibitory effects of the plant extracts indicate the presence of phytotoxic substances. The extracts in this study were subjected to chromatographic fractionations, and two substances were isolated and characterized using spectral analysis as vanillic acid and gallic acid. These two substances are phenolic compounds. Phenolic substances are the most common classes of phytochemicals that have important morphophysiological significance in various plants and exhibit various biological activities like anti-inflammatory, antimicrobial, and antioxidant effects [34]. Phenolic substances are the most significant phytochemicals that are involved in allelopathy [35,36]. The development of secondary metabolites, particularly phenolic substances, is important for the survival of plants and help to improve their self-defense and plant protection [37].

Vanillic acid is present in various fruits, cereal grains, olives, and different plants, along with beer, cider, wine [38,39], *Gardeniae fructus* [40], potato [41], red propolis [42], palm plant [43], *Juglans regia* [44], *Angelica sinensis* [45,46], *Chenopodium murale* [47], pumpkin seeds [48], *Melilotus messanensis* [49], orchard grass [50], and *Poliomintha longiflora* [51]. Vanillic acid is commonly used in different food preservatives, additives and flavoring agents and in the perfume industry [52]. It also plays an important role in protein and fatty acid biosynthesis [53]. Vanillic acid is applied as an antibacterial agent [54,55]; is a natural antioxidant in vegetables, fruits, and other plants [44,56]; is also used in antiapoptotic, hypotensive, hepatoprotective, cardioprotective roles and in the regulation of genes [57,58,59]. It is also commonly used in prescriptive Chinese drug [60]. Although vanillic acid has been identified in different plants and its allelopathic activity is well documented [61,62,63,64,65], this is the first report on vanillic acid from *Wedelia chinensis*.

Gallic acid is present in fruits and vegetables [66]; gallnuts, grapes, and blackberries [67]; eucalyptus species and *Picea schrenkiana* [68]; tea [69]; chestnuts and several berries [51]; *Myriophyllum spicatum*, *Cynomorium coccineum*, and *Microcystis aeruginosa* [70,71]; and black tea [72]. It has many medicinal uses such as antimicrobial [73], neuroprotection [74], antioxidant [75,76], anticancer [77,78], and antiulcer [79] uses and for cardiovascular diseases (CVDs) [80]. Furthermore, gallic acid has lipid homeostasis [81] and antihyperglycemic effects [82] and acts as a cardioprotective agent [83,84]. Gallic acid is also used in the food industry; in manufacturing inks, paints, and dyes; and in cinematography [85]. Gallic acid has been identified in various plants, and its allelopathic potential has also been shown in many studies [86,87,88,89], but this is the first report on gallic acid in *Wedelia chinensis* so far.

The I_50_ values show that the inhibitory activity of vanillic acid against the growth of cress and Italian ryegrass was stronger than that of gallic acid. The disparity in phytotoxic activity might be due to the difference between their chemical structures as the phytotoxic activity of allelopathic compounds is determined based on their structural variations [90,91]. Vanillic acid and gallic acid (a derivative of benzoic acid) contain a benzene ring. In vanillic acid, an OH group, an OCH_3_ group, and a COOH group are found on the benzene ring, and gallic acid has three OH groups and one COOH group but no OCH_3_ group. Research on the relationships between structure and activity showed that the number and location of OH and OCH_3_ groups determine the phytotoxic effects of benzoic acid [92]. In addition, hydroxy and methoxy substituents in the benzene ring have been reported to either decrease or increase the phytotoxic effects of benzoic acid [93]. However, a compound to drug should have amphipathic characteristics, including aqueous solubility and sufficient lipid solubility. Vanillic acid and gallic acid both have excellent aqueous solubility due to their COOH group. However, the methoxy group in vanillic acid increases more lipid solubility than that of the hydroxyl group present in gallic acid, which may be the reason that vanillic acid shows stronger inhibitory effects compared to the gallic acid.

Therefore, the growth inhibitory potential of vanillic acid and gallic acid lead to the phytotoxicity of *Wedelia chinensis*. Hence, the phytotoxic effects of *Wedelia chinensis* allow this plant to develop vigorously with minimal weed infestation because it inhibits nearby plants by releasing allelopathic substances.

## 4. Conclusions

The obtained results from the study suggested that *Wedelia chinensis* possesses potent phytotoxic effects, repressing the growth of lettuce, alfalfa, cress, rapeseed, barnyard grass, Italian ryegrass, timothy, and foxtail fescue. Two phytotoxic compounds, vanillic acid and gallic acid, were isolated from the aqueous methanol extracts of *Wedelia chinensis*. Vanillic acid and gallic acid significantly impeded the growth of cress and Italian ryegrass seedlings. This is the first report on the phytotoxic activity of *Wedelia chinensis*. Our research indicated that *Wedelia chinensis* can be used for the biological control of weeds, which may help to develop organic farming.

## Figures and Tables

**Table 1:** The I_50_ values of the aqueous methanol extracts of *Wedelia chinensis* against shoot and root growth of the tested species.

Tested Species		I_50_ Values (mg DW Equivalent Extract/mL)	
		Shoot	Root
Dicot	Alfalfa	32.6	48.0
	Cress	7.8	13.9
	Lettuce	3.3	8.7
	Rapeseed	8.9	13.1
Monocot	Barnyard grass	42.2	16.4
	Foxtail fescue	21.3	17.3
	Italian ryegrass	24.7	23.9
	Timothy	17.1	10.4

**Table 2:** The I_50_ values of vanillic acid and gallic acid from *Wedelia chinensis* against the shoot and root growth of cress and Italian ryegrass.

Tested Species		Vanillic Acid	Gallic Acid
		(mM)	
Cress	Shoot	0.04	15.4
	Root	0.05	13.8
Italian ryegrass	Shoot	0.47	6.6
	Root	0.45	2.3
